# Frontal Brain Activity and Behavioral Indicators of Affective States are Weakly Affected by Thermal Stimuli in Sheep Living in Different Housing Conditions

**DOI:** 10.3389/fvets.2015.00009

**Published:** 2015-05-12

**Authors:** Sabine Vögeli, Martin Wolf, Beat Wechsler, Lorenz Gygax

**Affiliations:** ^1^Centre for Proper Housing of Ruminants and Pigs, Federal Food Safety and Veterinary Office FSVO, Agroscope, Institute of Livestock Sciences ILS, Ettenhausen, Switzerland; ^2^Animal Behaviour, Institute of Evolutionary Biology and Environmental Studies, University of Zurich, Zurich, Switzerland; ^3^Biomedical Optics Research Laboratory, Division of Neonatology, University Hospital Zurich, Zurich, Switzerland

**Keywords:** temperature, emotion, mood, housing conditions, functional near-infrared spectroscopy, sheep

## Abstract

Many stimuli evoke short-term emotional reactions. These reactions may play an important role in assessing how a subject perceives a stimulus. Additionally, long-term mood may modulate the emotional reactions but it is still unclear in what way. The question seems to be important in terms of animal welfare, as a negative mood may taint emotional reactions. In the present study with sheep, we investigated the effects of thermal stimuli on emotional reactions and the potential modulating effect of mood induced by manipulations of the housing conditions. We assume that unpredictable, stimulus-poor conditions lead to a negative and predictable, stimulus-rich conditions to a positive mood state. The thermal stimuli were applied to the upper breast during warm ambient temperatures: hot (as presumably negative), intermediate, and cold (as presumably positive). We recorded cortical activity by functional near-infrared spectroscopy, restlessness behavior (e.g., locomotor activity, aversive behaviors), and ear postures as indicators of emotional reactions. The strongest hemodynamic reaction was found during a stimulus of intermediate valence independent of the animal’s housing conditions, whereas locomotor activity, ear movements, and aversive behaviors were seen most in sheep from the unpredictable, stimulus-poor housing conditions, independent of stimulus valence. We conclude that, sheep perceived the thermal stimuli and differentiated between some of them. An adequate interpretation of the neuronal activity pattern remains difficult, though. The effects of housing conditions were small indicating that the induction of mood was only modestly efficacious. Therefore, a modulating effect of mood on the emotional reaction was not found.

## Introduction

Research on affective states, that is, short-term emotions (1) and long-term mood (2) is of interest because of their basic influence on behavior in humans and other animals. Emotions are often seen as the concerted physiological, behavioral, and cognitive reaction of a subject (1). Emotions can be characterized by their valence ranging from negative to positive and by their arousal ranging from low to high, as described by dimensional models of emotion (2). Valence and arousal depend on the stimulus eliciting the emotion and reflect how the stimulus is perceived by the subject (2).

In our previous studies with sheep (3, 4), we investigated physical and social stimuli that varied in their presumed valence to examine the emotional reactions of sheep. It seemed that the sensory channel, that is, physical touch (3) or video images (4) through which the sheep experienced the stimuli influenced their reactions. A further sensory channel is the sense of temperature and temperature seems to have an important impact on sheep. Sheep, which are exposed to hot ambient temperatures, may experience heat stress reflected by metabolic changes [e.g., heart rate (5), evaporation (6), and respiratory rate (7)]. Also, Wojtas et al. (8) found that sheep under heat stress conditions reacted to air movements with a reduction in heart and respiratory rate indicating a reduction in heat stress. If temperature is very high, pain may be induced at least in humans (9).

How a stimulus is perceived can be communicated verbally by humans, but non-verbal indicators are needed in animals (1, 10). In previous studies with sheep, different behaviors were used as indicators of emotional valence: locomotor activity, aversive behavior (3), escape behavior such as jumping/rearing up the walls (3, 11), ear movements, specific ear postures [e.g., Ref. (3, 12–14)], and rumination (15, 16). Muehlemann et al. (17) introduced functional near-infrared spectroscopy (fNIRS) to measure changes in hemodynamic reactions reflecting brain activity in the frontal cortex as an additional indicator for emotional reactions in sheep. Stimuli that evoke a neuronal activation lead to changes in oxy- and deoxyhemoglobin concentrations in the brain, which can then be measured by fNIRS. This method focuses on the brain area where emotions are processed (18, 19) and therefore is a promising indicator for measuring emotions.

Apart from short-term emotional reactions, long-term mood is a second important aspect in the characterization of affective states. Long-term mood seems to modulate short-term emotional reactions (20). Studies in clinically depressed human subjects showed that a negative mood state makes the emotional reaction toward negative events more and toward positive events less extreme (21). This means that depression coincides with a general tainting of emotional experiences, that is, all emotional reactions are more negative compared to non-depressed controls. In animals, Reefmann et al. (22) found a different effect of mood, in that positive mood seemed to have a stabilizing effect on emotional reactions. In that experiment, sheep in a more positive mood reacted more weakly to negative as well as positive stimuli. Effects of mood on short-term emotions are specifically relevant for animals in terms of animal welfare because negative mood may taint all emotional experiences and positive mood may weaken the effect of short-term negative experiences. However, detailed studies on these hypotheses, that is, general tainting by a relatively more negative mood or a stabilizing effect of emotional reactions by a more positive mood are still missing.

Mood is thought to develop by an accumulation of either negative or positive events (2), which leads to a relatively more negative or positive mood state, respectively. Differences in housing conditions have been found to be associated with such positive or negative events. For example, unpredictability of the housing conditions [e.g., Ref. (23, 24)] or a stimulus-poor, barren environment [e.g., Ref. (25)] can induce a negative mood state. Elements of enrichment like, for example, increased space allowance or the addition of new resources have been shown to induce a relatively more positive mood [e.g., Ref. (26, 27)]. Mood can then directly be measured by the so called cognitive judgment bias test (3, 10, 28) or indirectly by its modulatory effect on emotional reactions (3, 4, 22).

In the present study, we extended the range of sensory channels through which emotional stimuli can be perceived by using thermal stimuli and investigated their effects on neurobiological and ethological measures of affective states in sheep. We assumed that sheep exposed to high-ambient temperatures would experience a cold temperature applied to the skin of their frontal trunk as positive, but would perceive heat as negative in the same situation. We chose a hot but not painful temperature to evoke an unpleasant feeling without eliciting pain. This is important because pain is also processed in the frontal cortex [reviewed for humans in Ref. (29)] and may elicit different reactions than an emotional reaction triggered by a thermal stimulus. Therefore, we chose stimulus temperatures that were not too far from to the sheep’s thermoneutral skin temperature, which was reported to be between 33 and 35°C (30). In addition, a temperature intermediate to the cold and hot stimulus was applied as a neutral emotional stimulus. In addition, we varied housing conditions to induce different mood states and elucidate the relation between mood state and emotional reaction. Sheep were kept under either unpredictable, stimulus-poor or predictable, stimulus-rich housing conditions. Data on neuronal activity based on near-infrared spectroscopy of the frontal cortical area, ear postures, locomotor activity, and restlessness behaviors were collected as indicators of an emotional reaction when sheep from two different housing conditions were confronted with thermal stimuli of different valence. The valence was assumed to be negative, intermediate, and positive for sheep kept at a warm ambient temperature when the temperatures applied were chosen as hot, intermediate, and cold, respectively. We hypothesized that the hot temperature would evoke the highest levels in neuronal and locomotor activity and the highest number of aversive behaviors and rearing up the walls, whereas the cold temperature would evoke ruminating and nibbling most frequently. The neuronal activity, locomotor activity, aversive behaviors, and rearing up the walls were additionally assumed to be reduced in animals from the predictable, stimulus-rich housing conditions, compared with the sheep from the unpredictable, stimulus-poor housing conditions, when confronted with the negative as well as the positive stimulus. This assumption was based on the observations of Reefmann et al. (22) that the positive mood stabilizes the animal’s reactions.

## Materials and Methods

### Animals and housing

The experiment was performed with 24 focal female, non-reproducing and non-gestating Lacaune sheep randomly chosen out of a group of 29 sheep of an average age of 2.5 years and an approximate average weight of 80 kg. To induce different mood states, the animals were housed in 2 pens with 14 and 15 sheep under unpredictable, stimulus-poor and predictable, stimulus-rich conditions, respectively, at Agroscope in Tänikon, Switzerland (3, 4, 28).

Sheep in the predictable, stimulus-rich housing conditions lived in an open-front pen of 58 m^2^ separated in a feeding (concrete floor) and lying area (deep litter straw) with additional structuring elements and an outdoor run area. Except during winter time, sheep were kept on pasture during the night (from April 2013 onward in the year of this experiment). Animals were fed regularly (in the current experiment daily between 07:30 and 08:00 h and between 16:30 and 17:00 h) with a diet of hay and a salt block (Kroni-385 Magnesia Natura), had access to water *ad libitum* and were provided with natural daylight (3, 4, 28).

The pen with the unpredictable, stimulus-poor housing conditions had an area of 22.4 m^2^. Moreover, the sheep in these conditions were exposed to an irregular daylight cycle (randomly varying between 6 and 16 h) and received food (hay) and water at unpredictable times [twice per day; Ref. (3, 4, 28)]. A salt block was also available at *ad libitum*. Sheep had been housed in these conditions for about 1.5 years (since July 2011), and the long-term effects of both housing conditions on the mood states were rather weak in an earlier assessment (3). Therefore, we attempted to intensify the effect of the unpredictable, stimulus-poor conditions in the current experiment. For 6 weeks (between early May and mid-June 2013), the unpredictable, stimulus-poor housing conditions were relaxed and sheep received food, water, and daylight regularly and were allowed to use an additional indoor run area in which they were confronted with environmental stimuli (28). Nine weeks before the actual experiment (mid-June 2013), the conditions were tightened again, that is, the animals experienced the unpredictable, stimulus-poor conditions in respect to food and daylight described above on the minimal area of the pen (3, 4, 28). Additionally, the group was now subdivided by a metal grid in two subgroups of seven sheep each, and the composition of those two groups was inter-changed every 2–5 days as an additional unpredictable factor (28).

### Stimuli and stimulus device

**Figure 1:**
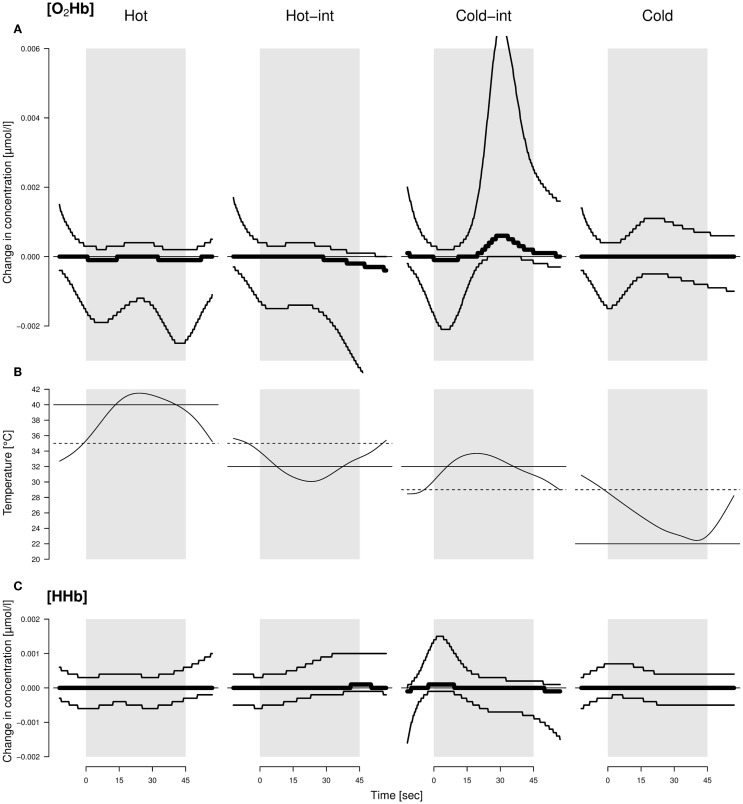
**Average changes in concentration of O_2_Hb (A) and HHb (C) throughout the application of different thermal stimuli (B) in sheep**. Hot-int (hot-intermediate) and cold-int (cold-intermediate) describe the direction of temperature change from the inter-stimulus to the stimulus phase. Black lines: models with highest model probabilities (O_2_Hb and HHb: the main effects stimulus valence as a factor and time as well as their interaction). Thin lines: 95% confidence intervals. See text for further information on the models. The best model did not include housing condition; hence, the displayed pattern was similar for both housing conditions. The average temperature profile per valence is displayed by the curves in the middle. Solid line: designated temperature for the stimulus; dotted line: designated temperature for the inter-stimulus phase.

To evoke an emotional reaction, sheep were exposed to thermal stimuli applied to their frontal trunk and varying in expected valence. As the experiment was run in the summer (August 2013) with average ambient temperature outside of 19.5 and 23.5°C in the test pen, cold temperature was presumed to be of positive, neutral temperature to be of intermediary, and hot temperature to be of negative valence. To provide the sheep with a constant warm ambient temperature during testing, two electronic infrared heaters (each 1.18 m × 0.07 m in size, 1,000 W each, 230 V; JO-EL Electric, Norway) were mounted 1.5 m above the test pen to keep the air temperature at an average of 25.5°C with additional radiant heat. All temperatures were applied to the animal by a mechanical device fixed by a harness at the front of the animal’s trunk. The device consisted of two Peltier-elements (PE-127-10-13, Farnell AG, Zug, Switzerland) fixed on a metal plate (80 cm × 60 cm) and a small ventilator on the backside for faster cooling. The device was connected with a wireless remote control (3) and controlled by the DasyLab software (11.0). The stimuli were applied to the animals in three phases: pre-stimulus, stimulus, and post-stimulus phase (see Measurements: Frontal Brain Activity). For the cold stimulus (cold), the temperature of the surface of the stimulus device was reduced from 29°C (pre-stimulus phase) to 22°C at the start of the stimulus and increased back to 29°C (post-stimulus phase) at the end of the stimulus (Figure 1B, right). During the pre- and post-stimulus phases (inter-stimulus phases), more neutral temperatures were applied to the sheep. For the hot stimulus (hot), the temperature was changed from 35 to 40°C (Figure 1B, left). For the neutral stimulus, a temperature of 32°C was chosen in the stimulus phase that was reached from either 29°C (cold-int) or 35°C (hot-int) in the inter-stimulus interval for each of half of the sheep per housing condition to balance for effects of increasing and decreasing temperatures (Figure 1B, two centered). Separate inter-stimulus temperatures were necessary to avoid extreme differences between the stimulus and the inter-stimulus temperature, in order to reduce the time needed for heating up and cooling down the device (for the average temperature curves, see Figure 1B). In humans, small changes in temperature applied directly to the subject’s skin were perceived as uncomfortable (personal observation) and therefore assumed to be uncomfortable for the animals as well.

The actual time and duration of the stimulus phase were calculated based on the effective temperature values of the stimulus device that had been recorded to the nearest degree continuously at 2 Hz throughout the measurements. The start of a switch between phases was scored at the time when the following criteria were met: (1) the first point in time when the designated temperature changed, that is, when the device started cooling or warming; (2) when the temperature had remained constant or changed in the direction from the pre-switch temperature to the post-switch temperature for three continuously recorded values (i.e., over a time span of 6 s); and (3) when the temperature changed toward the post-switch temperature by at least 2°C. For the analyses, the pre- and post-stimulus phases were cut at 12 s each. The actual stimulus duration varied from stimulus to stimulus because the time needed for heating or cooling was variable. In the analyses of the fNIRS measurements, the lengths of the stimulus phases were adjusted to 45 s each, by stretching or shrinking the actual stimulus lengths by a factor of at most 3/2 or 2/3, respectively. Seventy-eight percent of the stimuli were adjusted with a factor of between 1.29 and 1. The adjustment was performed by means of spline interpolation (interp1 in R package signal) (31). For the analyses of the ear tracking data, no such length adjustment was necessary because the measures were calculated proportional to the actual stimulus length.

### Habituation and experimental procedure

The habituation started 5 weeks before the actual experiment and lasted from 15 to 31 July 2013. After habituation, the sheep were left undisturbed in their housing conditions for 2 weeks before the start of the actual test phase lasting 4 days. With the exception of one animal, the sheep were well habituated to the measurement equipment, as they had been tested with this equipment in previous experiments (3, 4). Nevertheless, a re-habituation to the fNIRS measurement equipment and the ear tracking targets (see Ethical Note) was conducted on two successive days before the start of the present experiment in the test pen with single sheep. After this habituation, the sheep did not show any behavioral signs after being equipped. For another 6 days, sheep were additionally habituated to the thermal stimuli to avoid a reaction purely based on novelty in the actual experiment. Each temperature was used twice for habituation with one temperature used per day: the intermediate temperature was applied twice for 5 min, the cold and hot temperatures for 5 and 10 min each on the first and second day, respectively.

Two days before the experiment, the tested animals were shaved on the head for close skin contact of the fNIRS sensor and on the upper breast for a direct contact of the temperature device using a hand-shearing machine and scissors. The head was additionally epilated using a human epilation cream on the eve of the experiment to avoid any interference between hair and the fNIRS sensor (light-piping). Epilation could lead to inflammation though this was not observed. Even if a rare subclinical inflammation should have occurred, this would have added to the inter-individual variability in our data set but would not have influenced measurements in respect to the different temperatures of the stimuli because these were tested within a short time period (see below).

The test pen measured 2 m × 2.5 m and was set up in the corner of a building with 1.2 m high walls on the non-corner sides. It was at <10 m straight line distance from both of the home pens and the experimental sheep could potentially hear their group mates. Sheep were led and if necessary gently driven from their home to the test pen in usually <3 min.

Sheep were led singly to the test pen where they were equipped with the measurement instruments and the electronic stimulus device. After a 5-min acclimatization period, the experiment began with a pre-stimulus phase of 30 s and was then followed by a stimulus and post-stimulus phase. This succession of pre-, stimulus, and post-stimulus phase was repeated a total of seven times. The stimulus phase, that is, the duration from the first change of the device’s designated temperature toward the stimulus temperature until the first change back to the inter-stimulus temperature lasted 45 s. The post- and pre-stimulus phases of the last and the current stimulus formed an inter-stimulus phase and lasted a random period of between 30 and 40 s chosen from a uniform distribution. This random variation was chosen to avoid anticipation of the next stimulus. Each valence of the stimulus was repeated seven times per sheep and all three valences were applied to the animal one after the other on the same day. The order of the valences was balanced between the sheep from the same housing conditions. Three animals of one of the two housing conditions were tested in the morning and three of the other in the afternoon. That is, a total of six animals were tested per day. Each test day started alternating sheep from one or the other housing conditions. As 24 sheep were tested on 4 days, 6 animals of each housing condition were tested in the morning and 6 in the afternoon. During the time in the test pen, a familiar experimenter was in the test pen with the animal, but did not interact with the sheep. This experimenter was blinded to the stimulus sequence.

### Measurements: Frontal brain activity

The thermal stimuli were supposed to evoke a neuronal activation in the animals, which can be detected as a change of the oxy- and deoxyhemoglobin concentrations ([O_2_Hb] and [HHb], respectively) in the brain, measurable by fNIRS (17). The fNIRS sensor (7.0 cm × 3.4 cm) with two different wavelengths (LED at 760 and 870 nm) used in this study was placed on the top of the sheep’s head, at the most frontal part possible for measuring the hemodynamic changes in the frontal cerebral cortex. The measurement area was therefore positioned approximately from the central sulcus forward on the midline of the head. The alignment of the four light sources and the two detectors on the wireless sensor allowed measuring potential location effects (right–left, caudal–cranial, deep–shallow) of the neuronal activity (32). The recorded sampling rate of 100 Hz was reduced to 1 Hz for analyses of [O_2_Hb] and [HHb] (3, 4, 28).

### Measurements: Restlessness behavior and ear postures

Several restlessness behaviors of the animals were registered throughout the seven repetitions of each stimulus according to previous studies (3, 4): (1) the number of aversive behaviors (shaking, stamping, bucking, and moving backwards), (2) the number of times rearing up the walls, (3) the number of times nibbling the accompanying person, and (20) (4) ruminating (number of repeated stimuli during which sheep ruminated, at most seven). The recording of these behaviors was done directly by two observers. Each of the two observers recorded two behaviors using handheld tally counters (HC-2, Voltkraft, Hirschau, Germany). The two observers recorded different behavioral patterns, that is, they recorded the same behavior for all tested animals. As the three phases per sheep with the seven repetitions of each stimulus had the same length, absolute numbers of observed behaviors were compared.

Ear postures and movements as indicators of emotions (3, 4, 12–14) were measured by an automatic tracking system (TrackPack4, Advanced Realtime Tracking GmbH, Weilheim, Germany) consisting of four infrared cameras located in the four top corners of the test pen, a head target (four connected reflective marker balls fixed by a halter on the top of the head between the ears, 142 g), and two single reflective marker balls [fixed by a screw on the backside of the earmarks of both ears, 2.6 g; for more detailed information see Ref. (33)]. We distinguished between four different ear postures, all calculated as a proportion of time and for each single repetition of the stimuli: forward ears (both ears pointed more than 0 degrees forward), backward ears (both ears pointed more than 10 horizontal degrees backward), passive ears (both ears more than 30 vertical degrees below horizontal), and left ear forward (left ear positioned more than 5 horizontal degrees more forward than right ear per all ear positions with more than 5 degrees difference in their horizontal angle). In addition, ear movements were measured as the sum of the absolute differences between successive horizontal angles of both ears divided by the duration of phase (degrees/s). The tracking system was additionally used for measuring the locomotor activity of the sheep during the pre-stimulus, stimulus, and post-stimulus phases as the cumulative distance covered by the head target divided by the duration of phase (m/s). This locomotor activity included both, head movements through space (because the body was moved) and head movements relative to the body.

### Statistical evaluation

The evaluations of the brain and behavior data were all performed with the package lme4 (34) implementing linear mixed-effects models in R (version 3.01) (35). Graphical analyses of residuals were performed in respect to model assumptions and choice of transformations. The data of locomotor activity, ear movements, and restlessness behavior were log transformed, ear posture data were logit transformed and data of [O_2_Hb] and [HHb] were transformed according to Gygax et al. (36). The final model was selected by ranking model probabilities (mPr) based on the Bayesian information criterion in a given set of models (BIC) (36). Apart from the fNIRS models, we included all possible models ranging between a maximum model as defined below and the minimal model, which included a constant and corresponds to the notion that none of the considered fixed effects had a systematic influence on the outcome variable in question. For all outcome variables, we included each model with stimulus temperature either coded as a factor or coded linearly in the set of evaluated models to be able to pin-point whether a given relationship of an outcome variable with temperature is linear or not. The maximum model of the restlessness behaviors included the fixed effects housing condition (factor with two levels: predictable and unpredictable) and temperature of the stimulus (factor with four levels: cold, cold-int, hot-int, hot) and the interaction between the fixed effects. The maximum models of the locomotor activity, ear movements, and ear postures included the additional fixed effect phase (factor with three levels: pre-stimulus, stimulus, and post-stimulus) and all potential interactions between fixed effects. The random effect in the analyses of the restlessness behaviors was the sheep’s identity (coded as a factor). The other behavioral analyses included the stimulus repetition nested in period with a given stimulus temperature nested in animal identity as the random effect (all coded as factors). The temperature of the stimulus was coded either as a linear term or as a factor to test for a non-linear relationship between the four types of stimulus temperature.

For the analysis of the fNIRS data, the values were averaged over 3 s to correct for a restriction of lmer in modeling temporal correlations (3, 17, 36). This resulted in 92,736 potential observations: 24 sheep × 3 types of stimulus temperature [cold, int, hot] × 7 repetitions × up to 8 light paths × 23 values throughout each repetition. Of those, 90,664 observations were analyzed, which corresponds to 97.8% of the potential observations. Some stimuli and paths had to be excluded because of movement artifacts or extreme deviations of effective stimulus length from default stimulus length.

For the evaluation of the fNIRS data, the maximum model included all fixed effects of housing condition (factor with two levels), stimulus temperature (factor with four levels), time course (i.e., the time elapsed from the pre-stimulus to the stimulus, and the post-stimulus phase: natural spline function of a continuous variable), side (factor with two levels: right and left), longitudinal position (factor with two levels: caudal, cranial), and measurement depth (factor with two levels: superficial, deep). First, a selection of degrees of freedom for the spline modeling of the temporal course of the signal was conducted. The optimal number of degrees of freedom that was searched among the values 5, 9, or 13 degrees of freedom. The time course of [O_2_Hb] and [HHb] was best modeled by a natural spline with the smallest of the tested degrees of freedom (df = 5; both mPr = 1). Five degrees of freedom were then used in the following model selection including the simplest model (null model) up to the maximum model (all fixed effects and all potential interactions between the fixed effects), resulting in 33 models, all including the random effects of light path (corresponding to all eight possible combinations of side, longitudinal position, and measurement depth) nested in stimulus repetition nested in period with a given stimulus temperature nested in animal identity (all coded as factors).

## Results

### Brain activity

There was strong evidence that [O_2_Hb] and [HHb] were influenced by an interaction of stimulus temperature and time course (mPr = 1.00, evidence ratio in respect to the null model *E*_0_ = 1.6 × 10^38^ and mPr = 0.99, *E*_0_ = 148.4, respectively). [O_2_Hb] decreased in the first and increased in the second half of the cold-intermediate stimulus, whereas [HHb] increased at the beginning of the cold-intermediate stimulus (Figure 1). No consistent changes in [O_2_Hb] and [HHb] were observed for the hot, hot-intermediate, or cold stimulus (Figure 1). No differences were found between the housing conditions in change of [O_2_Hb] or [HHb].

### Behavioral indicators: Locomotor activity and ear postures

**Figure 2:**
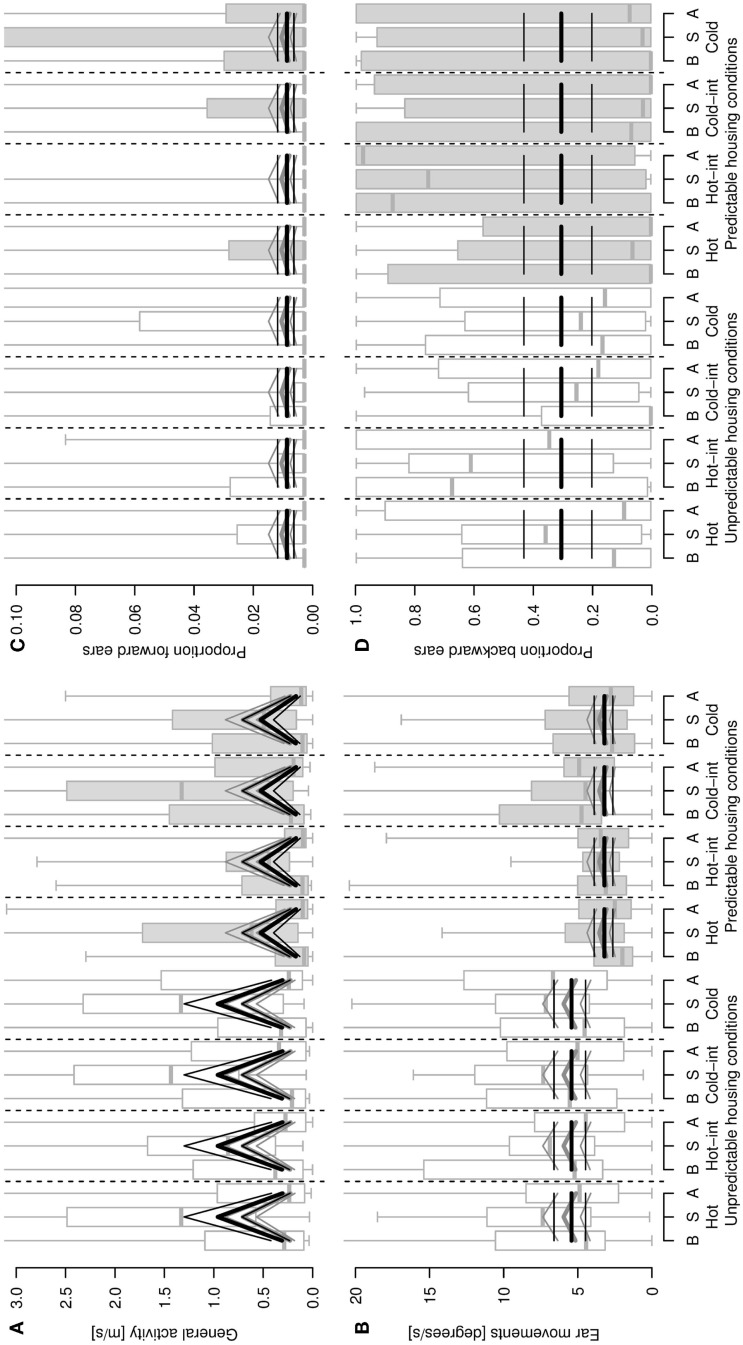
**General activity [distance covered, m/s, (A)], ear movements [degrees/s, (B)], proportion of forward (C) and backward (D) ear postures as a function of sheep’s housing condition (unpredictable, stimulus-poor and predictable, stimulus-rich), stimulus type and phase of the stimulus (B = before/pre-stimulus, S = stimulus, A = after/post-stimulus)**. Hot-int (hot-intermediate) and cold-int (cold-intermediate) describe the direction of temperature change from the inter-stimulus to the stimulus phase (see also Figure 1B). Box plots indicate data range as well as median, lower, and upper quartile. Thick black lines: model estimates with the highest model probabilities; gray lines: models with second-highest model probability; thin lines: 95% confidence intervals. *Y* -axes are cropped in **(A–D)** to enhance visibility of the pattern reflected by the statistical estimates.

Locomotor activity was elevated during the stimulus compared with the pre- and post-stimulus phases, and sheep living under unpredictable, stimulus-poor housing conditions exhibited a higher locomotor activity than the sheep living under predictable, stimulus-rich housing conditions (model including the main effects housing conditions and phase: mPr = 0.62, *E*_0_ ≫ 10,000; Figure 2A). The second-best model included the main effect phase only (mPr = 0.38, *E*_0_ ≫ 10,000). Similarly, with sheep from the unpredictable, stimulus-poor housing conditions moving their ears more often than sheep from the predictable, stimulus-rich housing conditions (mPr = 0.72, *E*_0_ = 3.87; Figure 2B). An additional effect of phase could only weakly be supported, but resulted in an increased amount of ear movements during the stimulus phase compared with the pre- and post-stimulus phases (model including housing conditions and phase: mPr = 0.08, *E*_0_ = 0.42). The proportion of forward ears was generally very low for both housing conditions, independent of stimulus temperature (best model including intercept only: mPr = 0.89; Figure 2C) and with a weak effect of the phase (mPr = 0.08, *E*_0_ = 0.09), whereas the proportion of backward ears was high during all temperatures irrespective of the housing conditions or phase (intercept only: mPr = 0.94; Figure 2D). No effects of the stimulus temperature, housing condition, or phase predictors were found for the proportions of passive ears (best model including intercept only: mPr = 0.97) and left ear forward (mPr = 0.97).

### Restlessness behaviors

Restlessness behaviors were shown at a low frequency throughout the testing procedure and some animals did not display some of the behaviors at all. The best models for all specific restlessness behaviors included the intercept only (aversive behavior: mPr = 0.71; rearing up the walls: mPr = 0.54; nibbling: mPr = 0.77; ruminating: mPr = 0.88). An effect of the housing conditions was detectable in each of the second-best models, that is, sheep from the unpredictable, stimulus-poor housing conditions showed more aversive behavior (mPr = 0.29, *E*_0_ = 0.41), rearing up the walls (mPr = 0.46, *E*_0_ = 0.85), and nibbling (mPr = 0.17, *E*_0_ = 0.22) as well as fewer stimulus repetitions with rumination (mPr = 0.12, *E*_0_ = 0.14) compared with the sheep from the predictable, stimulus-rich housing conditions.

## Discussion

The thermal stimuli used in this experiment had a general effect on the sheep’s behavior in that their locomotor activity and to a lesser extent their ear movements increased from the pre-stimulus to the stimulus phase and decreased from the stimulus to the post-stimulus phase. Therefore, the sheep seemed to perceive the temperature stimuli, and these stimuli elicited reactions that were almost as strong as those from a physical stimulus (3). Experiments in humans on the innocuous thermal detection threshold also showed that small changes in temperatures starting at 2°C were perceived (37).

If our stimuli would have changed from negative to positive as was assumed, we would have observed a graded reaction toward these stimuli in our measurements. Yet, we did not observe a monotonously graded reaction according to the temperature of the stimuli in our measures of restlessness behavior, locomotor activity, ear movements, ear postures, or brain activity. However, the temporal course of the hemodynamic signal varied with the thermal stimuli. Only in the intermediate condition where the temperature increased from 29 to 32°C (cold-int), a detectable increase in [HHb] at the beginning, a decrease in [O_2_Hb] at the beginning and a later increase in [O_2_Hb] in the second half of the stimulus phase was found. This can be interpreted as an initial deactivation and later activation of the frontal cortical area that we measured (38). This was unexpected because the temperature and change in temperature for that stimulus was moderate, that is, perceived as being within body temperature range when tested by a human, and therefore should not have prominently affected the animals. Coghill et al. (9) showed in humans that, during the stimulation with different temperatures ranging from 35°C up to over the pain threshold of 50°C, two regions of the prefrontal cortex were activated independent of the specific temperature. Additionally, the highest activation was found when the stimulus temperature was close to the pain threshold, and not during the application of the hottest temperature (9). That is the activation increased at pain threshold and then decreased. This would match the activation pattern found in the current study where the activation was highest with one of the intermediate stimuli instead of the highest temperature. However, the applied hot temperature in the present study was much lower than those used in the mentioned study with humans. It is therefore unlikely that one of the stimuli in the present study reached the sheep’s pain threshold and the experimental sheep would have perceived any pain. Based on a study of Hild et al. (39) with sheep, one also could argue that all the applied temperatures were not extreme enough to evoke any reaction in the animals. In that study, no behavioral reaction was observed below a temperature of 44°C. However, the applied temperatures used in the present study were chosen by pre-tests with non-focal animals from both housing conditions, and these animals had shown behavioral reactions even at smaller temperature differences than those used in the experiments. Again, a reaction during the intermediate stimulus would even be less expected if the stimuli had been so insignificant. Also, the stimulus temperature was exactly the same for both intermediate stimuli (hot-int and cold-int) and only the inter-stimulus temperatures were different. The observed brain activation, however, was only visible in the intermediate stimulus during which the temperature slightly increased (cold-int), and thus an explanation based purely on absolute temperatures seems difficult. In future studies, independent behavioral (choice) tests should additionally be conducted to be able to assess the animal’s perception of the stimulus valence more objectively and to aid interpretation.

Sheep of the two housing conditions behaved consistently differently during the experiments. Animals from the unpredictable, stimulus-poor housing conditions were more active in respect to locomotion and showed a higher amount of ear movements than the sheep from the predictable, stimulus-rich housing conditions. Given that these two outcome variables were log transformed in the statistical analyses, this difference was more pronounced in the stimulus than in the pre- and post-stimulus phases. A general difference and a more pronounced difference during the stimulus phase were also found in previous studies using physical and social stimuli, respectively (3, 4). As discussed by Vögeli et al. (3) and Guldimann et al. (28), this could be explained either by the challenge of the situation, which may have been more pronounced for the sheep from the unpredictable, stimulus-poor housing conditions (3, 28) in spite of the habituation procedure, or – less likely – by increased explorative behavior (40) provoked by the many stimuli arising during the testing procedure apart from the thermal stimuli. In parallel with the notion of an increased challenge due to the test situation, there was additional weak evidence that sheep from the unpredictable, stimulus-poor housing conditions exhibited more aversive behavior, reared up the walls more often and showed more nibbling but less ruminating than animals from the predictable, stimulus-rich housing conditions. Therefore, behavior patterns normally associated with negative situations such as fear [aversive behavior: (41, 42); raising up: (42, 43)] were displayed more by animals from the unpredictable, stimulus-poor housing conditions, and behaviors associated with positive situations [ruminating: (15, 16)] were displayed more by the animals from the predictable, stimulus-rich housing conditions. The increased locomotor activity and ear movements discussed above were therefore more likely linked with the challenge of the situation than with curiosity. Based on all these indicator variables, animals from the unpredictable, stimulus-poor housing conditions seemed to be more uncomfortable in the testing situation than animals from the predictable, stimulus-rich housing conditions, independent of the stimulus valence. This would be consistent with the hypothesis that the two housing conditions induced different mood states. As an additional indicator of mood states, a cognitive bias test was performed after the experiments described here, beginning with a training period that started 2 weeks after the current experiment, during which the sheep were again left undisturbed in their housing conditions. This cognitive judgment bias test showed that sheep from the unpredictable, stimulus-poor housing conditions were less likely and took longer to learn the testing paradigm but had, if only very weakly so, a positive judgment bias (28). The effects observed in the present study could thus result more directly from changes in how the sheep perceived thermal stimuli in general. It seems that any change in temperature applied directly to the body of the sheep was disturbing. This notion may be supported by the relatively high proportion of backward ears observed in the current study, an ear posture, which has often been associated with negative and stressful situations (12, 44, 45) although not previously been found in our own studies with other stimuli (3, 13).

Due to the only weak differences statistically assigned to the housing conditions, no modulating effect of the mood state on the emotional reaction to the thermal stimuli could be found.

## Conclusion

The present study showed that the thermal stimuli had a general effect on the sheep’s behavior, seen as an increased reaction during the stimulus phase compared with pre- and post-stimulus phases. Additionally, the sheep differentiated to some extent between the valences of the thermal stimuli. However, the strongest frontal brain activity occurred at an intermediate temperature contrary to our expectation of the most extreme reaction at an extreme temperature. At present and based on our data, an adequate interpretation of this pattern is not possible. Only a weak general effect of the housing conditions on the reaction of the animals was detected, and thus an induction of differential mood by the housing conditions seems moderately effective. Hence, we cannot state a potential modulating effect of the housing conditions on the reaction of the sheep to the different thermal stimuli.

## Ethical Note

All experiments were approved by the cantonal authorities (Canton of Thurgau, Switzerland, permit nos. F6/10 and F4/11) to conduct animal experiments. All animals at Agroscope, Tänikon, are considered farm animals and are kept according to the relevant regulations of the Swiss Animal Welfare law. The sheep used in the current experiment were purchased as lambs from two sheep dairy farms and they were used in several experiments of a larger project (3, 4, 28, 33). At the end of the project, the sheep were again sold to a working farm.

## Conflict of Interest Statement

The authors declare that the research was conducted in the absence of any commercial or financial relationships that could be construed as a potential conflict of interest.
